# Association of Functional Polymorphisms in *MSH3* and *IL-6* Pathway Genes with Different Types of Microsatellite Instability in Sporadic Colorectal Cancer

**DOI:** 10.3390/cancers16162916

**Published:** 2024-08-22

**Authors:** Anamarija Salar, Kristina Vuković Đerfi, Arijana Pačić, Anita Škrtić, Tamara Cacev, Sanja Kapitanović

**Affiliations:** 1 Laboratory for Personalized Medicine, Division of Molecular Medicine, Rudjer Boskovic Institute, 10000 Zagreb, Croatia; salar.anamarija@gmail.com (A.S.); kristina.vukovic@irb.hr (K.V.Đ.); 2 Department of Pathology and Cytology, University Hospital Dubrava, 10000 Zagreb, Croatia; arijanapacic@yahoo.com; 3 Department of Pathology and Cytology, University Hospital Merkur, 10000 Zagreb, Croatia; skrtic.anita@gmail.com

**Keywords:** microsatellite instability, MSH3, interleukin-6, gp130, interleukin-6 receptor, colorectal cancer

## Abstract

**Simple Summary:**

Microsatellite instability (MSI) is crucial in colorectal cancer (CRC) due to deficient mismatch repair (MMR), often caused by MLH1 and MSH2 loss of function. This defect affects microsatellite loci globally. Recently, MSI at tetranucleotide loci has been identified as a distinct entity, potentially arising from isolated MSH3 loss of function due to its translocation to the cytoplasm under interleukin-6 (IL-6) influence. This study examined the impact of *MSH3* and *IL-6* signaling pathway polymorphisms (*MSH3* exon 1, *MSH3+3133A/G*, *IL-6-174G/C*, *IL-6R+48892A/C*, and *gp130+148G/C*) on MSI types in sporadic CRC. A significant difference in *gp130+148G/C* genotypes (*p* = 0.037) and alleles (*p* = 0.031) was found, with the *C* allele being less common in tumors with di- and tetranucleotide instability (related to isolated MSH3 loss of function) compared to tumors without microsatellite instability. A functional polymorphism in *gp130* may influence the IL-6 pathway, leading to MSI linked to MSH3 loss of function.

**Abstract:**

Microsatellite instability (MSI) has been recognized as an important factor in colorectal cancer (CRC). It arises due to deficient mismatch repair (MMR), mostly attributed to MLH1 and MSH2 loss of function leading to a global MMR defect affecting mononucleotide and longer microsatellite loci. Recently, microsatellite instability at tetranucleotide loci, independent of the global MMR defect context, has been suggested to represent a distinct entity with possibly different consequences for tumorigenesis. It arises as a result of an isolated MSH3 loss of function due to its translocation from the nucleus to the cytoplasm under the influence of interleukin-6 (IL-6). In this study the influence of MSH3 and IL-6 signaling pathway polymorphisms (*MSH3* exon 1, *MSH3+3133A/G*, *IL-6-174G/C*, *IL-6R+48892A/C*, and *gp130+148G/C*) on the occurrence of different types of microsatellite instability in sporadic CRC was examined by PCR–RFLP and real-time PCR SNP analyses. A significant difference in distribution of *gp130+148G/C* genotypes (*p* = 0.037) and alleles (*p* = 0.031) was observed in CRC patients with the *C* allele being less common in tumors with di- and tetranucleotide instability (isolated MSH3 loss of function) compared to tumors without microsatellite instability. A functional polymorphism in gp130 might modulate the IL-6 signaling pathway, directing it toward the occurrence of microsatellite instability corresponding to the IL-6-mediated MSH3 loss of function.

## 1. Introduction

Colorectal cancer (CRC) is the third most common cancer and one of the leading causes of death in the Western world [1]. Approximately one-third of CRCs show familial clustering, but only up to 16% are linked to known germline pathogenic variants in CRC-predisposition genes. However, the majority of CRCs are sporadic, occurring without a known familial or hereditary link. They arise due to the multistep accumulation of acquired mutations and thus underscore the importance of lifestyle and environmental factors in tumorigenesis [2].

Microsatellite instability (MSI) arises due to the loss of function of mismatch repair (MMR) proteins. The loss of MSH2, MLH1, or PMS2 function results in complete absence of MMR, i.e., a global MMR defect causing instability at mononucleotide and longer microsatellite repeats. Isolated loss of MSH3 function results in instability at dinucleotide or longer repeats (but not at mononucleotides), while the MSH6 loss of function is associated with the instability at mono- and dinucleotide microsatellite sequences [3].

Elevated microsatellite alterations at selected tetranucleotide repeats (EMAST) has been proposed to represent a distinct type of microsatellite instability with possibly different consequences for tumorigenesis [3,4]. It can arise in the context of complete MMR disruption (global MMR defect) attributed to the MLH1 and MSH2 loss of function, but in recent years, another molecular mechanism leading to EMAST has been proposed. Haugen et al. [5] were the first to demonstrate that isolated hMSH3 loss of function is associated with the presence of EMAST in colorectal cancer cell line HCT116. Furthermore, they have also shown that cells deficient in MSH3 display a low level of microsatellite instability at dinucleotide repeats and an absence of instability at mononucleotide repeats. Further evidence of such an isolated MSH3 loss of function came from studies of families harboring rare biallelic *MSH3* germline mutations. Within these families, individuals carrying biallelic *MSH3* mutations exhibited microsatellite instability at di- and tetranucleotide loci, but not at mononucleotide loci in adenomas [6]. Consequently, the presence or absence of mononucleotide instability may serve as a discriminative marker for distinguishing between these two etiopathological molecular mechanisms leading to EMAST.

Since *MSH3* mutations or epigenetic silencing have not been predominantly reported as mechanisms of MSH3 inactivation in tumors, a model involving IL-6-mediated MSH3 translocation from the nucleus to the cytoplasm leading to an isolated MSH3 loss of function was recently proposed [7,8].

In addition, a number of MSH3 polymorphisms have been identified, yet only a few are hypothesized to potentially influence the function of the MSH3 protein. Among others, a polymorphic repetitive sequence consisting of nine base pairs (G[C]CCG[C]CAGCC) in the *MSH3* exon 1 was described [9]. Translation of this sequence into a protein results in a proline- and alanine-rich region called the poly-Ala tract. Tseng-Rogenski et al. [10] have shown that this region is located near the site that codes for the nuclear localization signal (NLS), which directs the MSH3 protein to the nucleus. Furthermore, the Δ allele, characterized by three fewer repeats compared to the full-length (FL) allele, results in a truncated poly-Ala tract. This shifts the NLS1 sequence to a less accessible position within the protein conformation, thereby hindering the re-entry of the MSH3 protein into the nucleus. Another *MSH3* functional polymorphism, *+3133A/G* (rs26279), associated with a higher predisposition to colorectal cancer, near the ATP binding site within the ATPase domain, has also been identified [11].

IL-6 signal transduction is mediated through either the classic or trans-signaling pathway, based on the presence or absence of the IL-6 receptor (IL-6R) at the cell surface. Since only few cell types have the IL-6R present in their membranes, most IL-6 signaling is trans- signaling, which has been implicated in tumorigenesis [12,13]. In the trans-signaling pathway, IL-6 binds to the soluble form of the IL-6R (sIL-6R), arising either from the alternative splicing of IL-6R mRNA or from the proteolytic cleavage of the ectodomain of membrane-bound IL-6R. For subsequent signal transduction, the coreceptor gp130, which is detected at the membrane of many different cells, including colon epithelial cells, is essential [14]. The coreceptor gp130 can also be found in the soluble form (sgp130), which binds to the IL-6/sIL-6R complex, thus preventing its interaction with the membrane-bound gp130. In this way the soluble coreceptor acts as an inhibitor of the trans-signaling pathway [15]. Consequently, the response of colon cells to IL-6 is modulated by the presence of the soluble IL-6R, membrane-bound or soluble forms of the gp130 coreceptor, and IL-6 levels.

The *IL-6* expression is affected by the presence of *IL-6* functional polymorphisms. One of the most frequently described polymorphisms affecting the expression of this cytokine is *IL-6-174G/C*, located in the promoter of this gene [16]. Jones et al. [17] have shown that elevated IL-6 plasma levels are detected in carriers of the C allele and CC genotype. Furthermore, a functional polymorphism located in the coding sequence of the interleukin-6 receptor, *IL-6R+48892A/C*, has also been associated with a twofold increase in the soluble IL-6 receptor [18]. Since this polymorphism is located near the ADAM17 cleavage site, it can potentially influence sIL-6R serum levels by modulating the IL-6R cleavage, which further leads to higher sIL-6R levels and increased IL-6 trans-signaling. Wonnereth et al. [19] were the first to investigate the impact of the *gp130+148G/C* polymorphism on the sgp130 levels in the serum of healthy individuals. They demonstrated that C allele carriers have higher levels of soluble gp130, which leads to a reduction in IL-6 trans-signaling.

Therefore, the aim of this study was to examine the possible influence of functional polymorphisms within the *MSH3* gene and *IL-6* signaling pathway genes (*gp130*, *IL-6*, and *IL-6R*) in individuals with sporadic colorectal cancer in the presence or absence of different types of microsatellite instability.

## 2. Materials and Methods

### 2.1. Patients

In our study, a cohort of 196 patients with sporadic colorectal cancer were analyzed. Specimens (tumor and adjacent normal colon tissue) from patients with sporadic colon cancer used in our study were obtained from the Croatian Tumor Bank at the Ruđer Bošković Institute, Zagreb, Croatia [20]. Ethics Committee approval was obtained and written consent was provided by all participants.

Demographic characteristics including age, sex, and family history of cancer were noted when samples for each individual were collected. The analyzed cohort consisted exclusively of patients with a negative family history of hereditary cancer. All specimens were obtained during routine surgery and none of the patients underwent preoperative irradiation or chemotherapy. Diagnoses were established by standard diagnostic procedures and confirmed by histopathology. Clinicopathological characteristics of colorectal cancer patients and their tumors are presented in Table 1. Fresh samples of resected colon carcinoma were snap-frozen in liquid nitrogen and stored at −80 °C until further use. DNA extraction was performed using a routine proteinase K digestion and phenol chloroform extraction protocol.

### 2.2. Microsatellite Instability Analysis

For the microsatellite instability analysis, paired tumors and normal DNA from the same patient were used and microsatellite instability was examined at two mononucleotide (BAT25, BAT26) and three dinucleotide (D2S123, D5S346, D17S250) loci according to the consensus of the NCI Workshop on Microsatellite Instability for Colorectal Cancer Detection [21]. To analyze microsatellite instability at tetranucleotide repeats, five additional markers (D20S85, D20S82, D9S242, D8S321, and MYCL1) were used. Analysis of two mononucleotide markers was performed using the ABIPRISM 310 genetic analyzer, with GeneMapper software, version 4.0 (Applied Biosystems, Foster, CA, USA). Dinucleotide and tetranucleotide markers were analyzed by polyacrylamide gel electrophoresis. Microsatellite instability was defined as either an alteration in length of the amplified PCR product (ladder pattern) or as a new band above or below that expected for the allele.

Tumors without microsatellite instability at any of the analyzed loci were considered stable (group S). Tumors exhibiting microsatellite instability were further classified in two groups to better discriminate between the global MMR defect and isolated MSH3 loss of function. Group E, corresponding to the isolated MSH3 loss of function, consisted of tumors in which microsatellite instability at one or more tetranucleotide loci was occurring independently or in association with instability at dinucleotide loci, but without instability at mononucleotide loci. Group M, corresponding to the global MMR defect, consisted of tumors in which microsatellite instability at mononucleotide loci was combined with instability at dinucleotide and/or tetranucleotide loci.

A total of 196 tumor samples were included in the study, of which, 115 were classified as stable (group S), 55 were classified as group E, and 26 tumors were classified as group M.

### 2.3. MSH3 Exon 1 Repeat Polymorphism Analysis

The *MSH3* exon 1 repeat polymorphism was assessed by PCR amplification, using oligonucleotide primers as follows: MSH3ex1F, 5′ TGA GCC GAT TCT TCC AGT CTA CGG G 3′ and MSH3ex1R, 5′ CCC AGT CCC AGA CAG AAC CTA CTA 3′. In brief, genomic DNA (100 ng) was used as a template in a reaction volume of 25 µL, containing 5 pmol of each primer, 50 µM of each dNTP, and 1 U of GoTaq DNA polymerase (Promega, USA). PCRs were carried out in an Applied Biosystems 2720 Thermal Cycler for 30 cycles with annealing temperature at 55 °C. Polymorphic marker analysis was performed by non-denaturing polyacrylamide gel electrophoresis followed by silver staining.

### 2.4. PCR and Restriction Fragment Length Polymorphism Analysis of the MSH3+3133A/G Polymorphism

PCR–restriction fragment length polymorphism (RFLP) analysis of *MSH3+3133A/G* (rs26279) was carried out as follows: PCR was performed using oligonucleotide primers as follows: MSH3 3133F, 5′ TTT CAG CTT TCA GGC ACA GTT 3′ and MSH3 3133R, 5′ CCT TCC AGC TCT TTT GAC TT 3′. In brief, genomic DNA (100 ng) was used as a template in a reaction volume of 25 µL, containing 5 pmol of each primer, 50 µM of each dNTP, and 1 U of GoTaq DNA polymerase (Promega, Madison, WS, USA). PCRs were carried out in an Applied Biosystems 2720 Thermal Cycler for 30 cycles with annealing temperature at 55 °C. For the RFLP analysis, 10 µL of the PCR product was digested overnight at 37 °C with 1 U of *Hha*I (New England BioLabs, Ipswich, MA, USA) at 37 °C in a volume of 25 µL. Samples were analyzed by agarose gel electrophoresis. Control samples covering three possible SNP genotypes and no template control were run in parallel with the tested samples in each experiment.

### 2.5. Real-Time PCR SNP Analysis of the IL-6-174G/C Promoter Polymorphism

Real-time PCR SNP analysis of *IL-6-174G/C* (rs1800795) was performed using an Applied Biosystems 7300 real-time PCR system and predeveloped TaqMan SNP genotyping assay c_1839697_20 (Applied Biosystems, Foster, CA, USA). The reaction mixture (25 µL) consisted of 100 ng of DNA, 1.25 µL of Taqman SNP Assay, 12.5 µL of TaqMan Universal PCR Master Mix (Applied Biosystems, Foster, CA, USA), and QH_2_O up to the final volume. Real-time PCR was carried out according to the manufacturer’s instructions and the results were analyzed using the 7300 System SDS Software, version 1.4 (Applied Biosystems, Foster, CA, USA). Control samples covering three possible SNP genotypes and no template control were run in parallel with the tested samples in each experiment.

### 2.6. PCR and Restriction Fragment Length Polymorphism Analysis of IL-6R+48892A/C and gp130+148G/C Polymorphisms

PCR–restriction fragment length polymorphism (RFLP) analysis of *IL-6R+48892A/C* (rs2228145) and *gp130+148G/C* (rs3729960) was carried out as described previously. Briefly, PCR was performed using oligonucleotide primers as follows: IL-6RF, 5′ CCT CTT TGT GCC TTG TG 3′ and IL-6RR, 5′ ATG GAT TAC CTC TTC GTG TC 3′; gp130F 5′ TGC CTC CAG AAA AAC CTA AA 3′ and gp130R 5′ CAT TCA GAT TTT AAA GTG AAG 3′. Genomic DNA (100 ng) was used as a template in a reaction volume of 25 µL, containing 5 pmol of each primer, 50 µM of each dNTP, and 1 U of GoTaq DNA polymerase (Promega, USA). PCRs were carried out in an Applied Biosystems 2720 Thermal Cycler for 30 cycles with annealing temperature at 52 °C. For the RFLP analysis, 10 µL of PCR product was digested overnight at 37 °C with 1 U of *Hinf*I (Promega, USA) or 1 U of *Mbo*I (New England BioLabs, USA) at 37 °C in a volume of 25 µL for *IL-6R+48892A/C* and *gp130+148G/C*, respectively. Samples of *IL-6R+48892A/C* and *gp130+148G/C* were analyzed by agarose gel electrophoresis and non-denaturing polyacrylamide gel electrophoresis, respectively. Control samples covering three possible SNP genotypes and no template control were run in parallel with the tested samples in each experiment.

### 2.7. Statistical Analysis

The Hardy–Weinberg equation was used to determine whether the proportion of each genotype obtained was in agreement with the expected values calculated from allele frequencies. Allele and genotype distributions between the S, E, and M groups were compared using Fisher’s exact test. A Fisher’s exact *p*-value < 0.05 was considered statistically significant. Homozygosity for the most common allele in Caucasians was used as the reference category. All evaluations were performed using the GraphPad Prism Software, version 8.0.2.

## 3. Results

### 3.1. Analysis of the MSH3 Exon 1 Polymorphism

The analysis of the 9 bp repetitive sequence in the first exon of the *MSH3* gene was performed using a polymerase chain reaction (PCR), followed by electrophoresis on a 10% non-denaturing polyacrylamide gel. The following alleles were observed: Δ (162 bp), del9 (180 bp), FL (189 bp), ins4 (193 bp), ins9 (198 bp), and ins18 (207 bp).

No statistically significant difference was observed in allele and genotype distribution with respect to the presence and type of microsatellite instability (Table 2). Nevertheless, changes in the MSH3 exon 1 polymorphism genotype were observed in tumor tissue compared to the corresponding normal mucosa, manifesting as loss of heterozygosity (LOH), which was observed in 27.4% (26/95) of the informative samples. The most common alteration observed in tumors was the loss of the Δ allele, resulting in a genotype change from Δ/FL to FL/FL, identified in 17 out of 26 (65.4%) informative tumors displaying allele loss. The most frequent alteration in tumors without the microsatellite instability (group S) was the loss of the Δ allele, present in 12 out of 19 (63.2%) samples. In the E group (isolated MSH3 loss of function), loss of the Δ allele was present in 5 out of 7 (71.4%) samples. In the M group (global MMR defect), no LOH was observed in any of the analyzed samples.

### 3.2. Analysis of the MSH3+3133A/G Polymorphism

PCR–RFLP was used to genotype the *MSH3+3133A/G* polymorphism. In brief, after the PCR amplification, PCR products (200 bp) were digested with HhaI in order to detect allele G (49 bp and 151 bp) and allele A (200 bp). No difference in allele or genotype distribution between the analyzed groups was observed (Table 3).

### 3.3. Analysis of IL-6-174G/C Promoter Polymorphism

Real-time PCR SNP analysis was used to genotype the *IL-6-174G/C* polymorphism in the promoter region of *IL-6*. Real-time PCR results were analyzed using the 7300 System SDS Software. The CC genotype was found to be more frequent in group E (isolated MSH3 loss of function) compared to group S; however, this difference was not statistically significant (*p* = 0.051). No difference in allele and genotype distribution was observed between the other analyzed groups (Table 3).

### 3.4. Analysis of the IL-6R+48892A/C Polymorphism

PCR–RFLP was used to genotype the *IL-6R+48892A/C* polymorphism. In brief, after the PCR amplification, PCR products (710 bp) were digested with *Hinf*I in order to detect allele A (66 bp, 74 bp, 239 bp, and 331 bp) and allele C (66 bp, 74 bp, and 570 bp). No difference in allele or genotype distribution between the analyzed groups was observed (Table 3).

### 3.5. Analysis of the gp130+148G/C Polymorphism

PCR–RFLP was used to genotype the *gp130+148G/C* polymorphism. In brief, after the PCR amplification, PCR products (120 bp) were digested with *Mbo*I in order to detect allele C (52 bp and 68 bp) and allele G (120 bp). A statistically significant difference in the distribution of genotypes (*p* = 0.037) and alleles (*p* = 0.031) between the E and S groups was observed. Both homozygous and heterozygous carriers of the C allele were less frequent in group E (isolated MSH3 loss of function) compared to group S. Furthermore, in group E, a reduced frequency of the C allele was observed when compared to group S. No difference in allele and genotype distribution was observed between the other analyzed groups (Table 3).

## 4. Discussion

Chronic inflammation and genome instability are some of the major contributing factors to tumorigenesis [22,23]. Interleukin-6 is one of the key mediators of both acute and chronic inflammation and its elevated levels in the serum and tumor tissue of colon cancer patients have been reported [24,25]. Loss of MLH1 and MSH2 function leads to a global MMR defect involving changes at mononucleotide and longer microsatellite loci. On the other hand, the isolated MSH3 loss of function leads to instability at dinucleotide and longer microsatellite repeats, but not at mononucleotides [5,26]. These findings are in agreement with MutSβ heterodimer function in preferential recognition of frameshift mutations ranging from 2–13 nucleotides [27].

The proposed mechanism for the isolated MSH3 loss of function is its translocation from the nucleus to the cytoplasm under the influence of IL-6. However, the exact molecular mechanisms underlying these processes have not yet been fully elucidated. It has been proposed that a 9 bp repeat polymorphism in the *MSH3* exon 1 could potentially impact the protein’s ability to translocate to the nucleus by affecting its stability, folding, interaction with MSH2, and recognition by nuclear transport mechanisms. Indeed, this repetitive sequence has recently been implicated in modulating the translocation of the MSH3 protein from the cytoplasm to the nucleus in response to treatment with IL-6. It has been shown in human colorectal cancer cell lines, specifically in HT29 carrying the full-length (FL) allele and SW480 harboring the deletion (Δ) allele, that treatment with IL-6 induces the relocation of the MSH3 protein. However, treatment with hydrogen peroxide, simulating conditions of oxidative stress, resulted in MSH3 protein translocation to the cytoplasm exclusively in cells carrying the Δ allele [8,10]. In our study, there was no difference in *MSH3* exon 1 allele and genotype distribution with respect to the presence and type of microsatellite instability. However, in the E group (isolated MSH3 loss of function), loss of the Δ allele was present in more than 70% of samples, while in the M group (global MMR defect), no LOH of the Δ allele was observed. Based on these findings, one might speculate that in the case of an isolated MSH3 loss of function, cells that have lost the Δ allele become less prone to MSH3 translocation triggered by oxidative stress. On the other hand, cells with a global MMR defect do not show the “evolutionary” preference for losing the ability to translocate MSH3 from the nucleus to the cytoplasm under the conditions of oxidative stress.

The net effect of the IL-6 trans-signaling pathway is determined by the presence and quantity of IL-6; its receptor, IL-6R, and coreceptor, gp130; as well as their soluble forms, sIL-6R and sgp130. Thus, in this study we have examined the possible influence of functional polymorphisms within the *IL-6*, *IL-6R*, and *gp130* genes in individuals with sporadic colorectal cancer on the occurrence of different types of microsatellite instability. In our study, we observed a higher frequency of *IL-6-174 CC* genotype carriers in group E compared to group S; however, this trend was not statistically significant (*p* = 0.051). It has been shown previously that the *IL-6-174 CC* genotype is associated with higher levels of IL-6 in the serum compared to the *GG* genotype [17]. Recent studies have also shown that chronic inflammation and IL-6-mediated MSH3 translocation from the nucleus to the cytoplasm are associated with the EMAST type of instability [8,10,28]. Therefore, the observation that *IL-6-174 CC* genotype carriers are more common in group E, in which EMAST occurs as a result of an isolated MSH3 dysfunction, is in line with previous findings.

The results of a meta-analysis have shown that for every inherited copy of the C allele, concentration of the IL-6R increases by approximately 34% and of IL-6 by 15% [29]. These findings might be interesting in relation to the previously mentioned net impact on the IL-6 trans-signaling and IL-6-mediated MSH3 translocation. However, in our study, there was no difference in the frequency of *IL-6R+48892A/C* alleles and genotypes among individuals with sporadic colorectal cancer with respect to different types of microsatellite instability.

To the best of our knowledge, this study is the first to examine the influence of *gp130+148G/C* polymorphism in sporadic colorectal cancer with respect to the presence of different types of microsatellite instability. The frequency of *C* allele carriers (genotypes *GC*+*CC*) was significantly lower in group E compared to group S (*p* = 0.037). Additionally, the frequency of the *C* allele was significantly lower in group E compared to group S (*p* = 0.031). Therefore, in this study, *C* allele carriers, who physiologically have elevated levels of sgp130, were less common in group E (isolated MSH3 loss of function) compared to those with tumors without microsatellite instability (group S). It has been shown that the fusion protein sgp130-Fc can inhibit inflammatory processes in the colon by blocking the IL-6 trans-signaling pathway in the animal model [30]. Additionally, Tseng-Rogenski et al. (2015) have shown that the presence of this fusion protein in colon cancer cell lines prevents the translocation of MSH3 from the nucleus to the cytoplasm following IL-6 treatment. Thus, one might speculate that the lower sgp130 levels in group E may create an environment that is favorable for signal transmission via the IL-6 trans-signaling pathway. On the other hand, potentially higher levels of sgp130 in group S could function as a buffer for IL-6, leading to reduced activation of IL-6 trans-signaling. Signal transmission through the IL-6 trans-signaling pathway is important in stimulating nearly every type of cell in the body, including intestinal epithelial cells, as well as tumor cells. Therefore, sgp130 potentially represents a crucial barrier that shields the cell from the possible negative effects of IL-6 [13].

Finally, we would like to address some limitations of the study. The sporadic CRC patients included in this study were selected based on their negative family history and were not sent for further germline mutation testing to confirm their sporadic status. Furthermore, patients and their tumors were not tested for MSH3 mutations. All specimens were obtained during routine surgery and none of the patients underwent preoperative irradiation or chemotherapy; however, we do not have any information about the patients’ treatment afterwards. Finally, our data set only included the Dukes’ stage of tumors and not the TNM stage.

## 5. Conclusions

Traditional MSI assessment primarily focuses on mononucleotide and dinucleotide repeats. However, elevated microsatellite alterations at selected tetranucleotide repeats (EMAST) can be driven by different molecular mechanisms compared to the conventional MSI. Besides being a part of a global MMR defect, this instability can also arise from the isolated dysfunction of MSH3 under the influence of IL-6. Since interleukin-6 plays a pivotal role in mediating both immune and inflammatory responses, another term, inflammation-associated microsatellite changes (IAMA), was also proposed for this type of instability. This term was coined to provide a better distinction between microsatellite instability due to IL-6-mediated MSH3 loss of function and global instability pattern linked to MLH1-induced MSH3 mutations in sporadic colorectal carcinomas, which can both present as EMAST when only tetranucleotide loci are examined [26]. Understanding the molecular development and progression of tumors with this type of instability could possibly lead to more effective and personalized diagnostic and therapeutic approaches. Nevertheless, further studies involving larger cohorts of patients are necessary to validate these findings.

## Figures and Tables

**Table 1:** Clinicopathological characteristics of colorectal cancer patients included in the study.

		*n* (%)	Group S (%)	Group M (%)	Group E (%)
Gender	M	121 (62.1)	71 (61.7)	14 (53.8)	36 (66.7)
	F	74 (37.9)	44 (38.3)	12 (46.2)	18 (33.3)
Age	≥65 y	127 (65.1)	79 (68.7)	15 (57.7)	33 (61.1)
	<65 y	68 (34.9)	36 (31.3)	11 (42.3)	21 (38.9)
Dukes	A	27 (13.8)	18 (15.7)	0 (0)	9 (16.7)
	B	75 (38.5)	41 (35.7)	14 (53.8)	20 (37.0)
	C	78 (40.0)	49 (42.6)	11 (42.3)	18 (33.3)
	D	15 (7.7)	7 (6.1)	1 (3.9)	7 (13.0)
Grade	1	62 (32.1)	37 (32.5)	9 (34.6)	16 (30.2)
	2	111 (57.5)	65 (57.0)	13 (50.0)	33 (62.3)
	3	20 (10.4)	12 (10.5)	4 (15.4)	4 (7.5)
Size	≥5 cm	106 (54.9)	54 (47.8)	20 (76.9)	32 (59.3)
	<5 cm	87 (45.1)	59 (52.2)	6 (23.1)	22 (40.7)

**Table 2:** Allele and genotype frequencies of the MSH3 exon 1 polymorphism in individuals with sporadic colorectal cancer with respect to presence and type of microsatellite instability.

MSH3 Exon 1	Group S	Group M	Group E
	*n* = 115	*n* = 26	*n* = 55
Genotype	*n* (%)	*n* (%)	*n* (%)
FL/FL	49 (42.6)	13 (50)	24 (43.6)
FL/∆	28 (24.3)	3 (11.5)	13 (23.6)
∆/∆	11 (9.6)	5 (19.2)	8 (14.5)
FL/ins9	11 (9.6)	3 (2.6)	5 (4.3)
FL/A5	3 (2.6)	1 (0.9)	1 (0.9)
FL/∆/ins9	4 (3.5)	0 (0)	0 (0)
∆/ins9	3 (2.6)	0 (0)	0 (0)
FL/ins18	0 (0)	0 (0)	3 (2.6)
ins9/ins9	2 (1.7)	0 (0)	1 (0.9)
FL/ins4	1 (0.9)	0 (0)	0
ins9/ins18	1 (0.9)	1 (0.9)	0
ins18/ins18	1 (0.9)	0 (0)	0
A5/∆	1 (0.9)	0 (0)	0
Alleles			
FL	145 (61.966)	33 (63.462)	33 (63.462)
∆	58 (24.786)	13 (25)	13 (25)
ins9	23 (9.829)	4 (7.692)	4 (7.692)
ins18	3 (0.013)	1 (0.004)	1 (0.004)
ins4	1 (0.004)	0	0
A5	4 (0.017)	1 (0.004)	1 (0.004)

**Table 3:** Allele and genotype frequencies of the *MSH3+3133A*/*G*, *IL-6-174G*/*C*, *IL-6R+48892A*/*C*, and *gp130+148G*/*C* polymorphisms in individuals with sporadic colorectal cancer with respect to presence and type of microsatellite instability.

Polymorphisms	Group S	Group E	*p*	Group S	Group M	*p*	Group E	Group M	*p*
	*n* = 115	*n* = 55		*n* = 115	*n* = 26		*n* = 55	*n* = 26	
*MSH3+3133A*/*G*									
Genotype	*n* (%)	*n* (%)		*n* (%)	*n* (%)		*n* (%)	*n* (%)	
AA	56 (48.7)	28 (50.9)	-	56 (48.7)	16 (61.5)	-	28 (50.9)	16 (61.5)	-
AG	52 (45.2)	27 (49.1)	>0.999	52 (45.2)	8 (30.8)	0.257	27 (49.1)	8 (30.8)	0.226
GG	7 (6.1)	0 (0)	0.095	7 (6.1)	2 (7.7)	>0.999	0 (0)	2 (7.7)	0.148
Alleles									
A	164 (71.3)	83 (75.5)	-	164 (71.3)	40 (76.9)	-	83 (75.5)	40 (76.9)	-
G	67 (28.7)	27 (24.5)	0.438	67 (28.7)	12 (23.1)	0.494	27 (24.5)	12 (23.1)	>0.999
*IL-6-174G*/*C*									
Genotype	*n* (%)	*n* (%)		*n* (%)	*n* (%)		*n* (%)	*n* (%)	
GG	37 (32.2)	16 (29)	-	37 (32.2)	8 (30.8)	-	16 (29)	8 (30.8)	-
GC	66 (57.4)	25 (45.5)	0.848	66 (57.4)	14 (53.8)	>0.999	25 (45.5)	14 (53.8)	>0.999
CC	12 (10.4)	14 (25.5)	0.051	12 (10.4)	4 (15.4)	0.715	14 (25.5)	4 (15.4)	0.506
Alleles									
G	140 (60.9)	57 (51.8)	-	140 (60.9)	30 (57.7)	-	57 (51.8)	30 (57.7)	-
C	90 (39.1)	53 (48.2)	0.127	90 (39.1)	22 (42.3)	0.754	53 (48.2)	22 (42.3)	0.505
*IL-6R+48892A*/*C*									
Genotype	*n* (%)	*n* (%)		*n* (%)	*n* (%)		*n* (%)	*n* (%)	
AA	48 (41.7)	23 (41.8)	-	48 (41.7)	9 (34.6)	-	23 (41.8)	9 (34.6)	-
AC	54 (47)	26 (47.2)	>0.999	54 (47)	17 (65.4)	0.278	26 (47.2)	17 (65.4)	0.336
CC	13 (11.3)	6 (11)	>0.999	13 (11.3)	0 (0)	0.193	6(11)	0 (0)	0.303
Alleles									
A	150 (65.2)	72 (65.5)	-	150 (65.2)	35 (67.3)	-	72 (65.5)	35 (67.3)	-
C	80 (34.8)	38 (34.5)	>0.999	80 (34.8)	17 (32.7)	0.872	38 (34.5)	17 (32.7)	0.860
*gp130+148G*/*C*									
Genotype	*n* (%)	*n* (%)		*n* (%)	*n* (%)		*n* (%)	*n* (%)	
GG	80 (69.6)	47 (85.5)	-	80 (69.6)	22 (84.6)	-	47 (85.5)	22 (84.6)	-
GC + CC	35 (30.4)	8 (14.5)	0.037 *	35 (30.4)	4 (15.4)	0.149	8 (14.5)	4 (15.4)	>0.999
Alleles									
G	191 (83)	101 (91.8)	-	191 (83)	47 (90.4)	-	101 (91.8)	47 (90.4)	-
C	39 (17)	9 (8.2)	0.031 *	39 (17)	5 (9.6)	0.212	9 (8.2)	5 (9.6)	0.769

*p*-values were calculated using the Fisher’s exact test; * *p* < 0.05.

## Data Availability

For the data generated or analyzed during this study that are not included in this article, inquiries can be directed to the corresponding author.

## References

[1] Bray F., Ferlay J., Soerjomataram I., Siegel R.L., Torre L.A., Jemal A. (2018). Global cancer statistics 2018: GLOBOCAN estimates of incidence and mortality worldwide for 36 cancers in 185 countries. CA Cancer J. Clin..

[2] Rebuzzi F., Ulivi P., Tedaldi G. (2023). Genetic Predisposition to Colorectal Cancer: How Many and Which Genes to Test?. Int. J. Mol. Sci..

[3] Carethers J.M. (2017). Microsatellite instability pathway and EMAST in colorectal Cancer. Curr. Color. Cancer Rep..

[4] Carethers J.M., Koi M., Tseng-Rogenski S.S. (2015). EMAST is a form of microsatellite instability that is initiated by inflammation and modulates colorectal cancer progression. Genes.

[5] Haugen A.C., Goel A., Yamada K., Marra G., Nguyen T.P., Nagasaka T., Kanazawa S., Koike J., Kikuchi Y., Zhong X. (2008). Genetic instability caused by loss of MutS homologue 3 in human colorectal cancer. Cancer Res..

[6] Adam R., Spier I., Zhao B., Kloth M., Marquez J., Hinrichsen I., Kirfel J., Tafazzoli A., Horpaopan S., Uhlhaas S. (2016). Exome Sequencing Identifies Biallelic MSH3 Germline Mutations as a Recessive Subtype of Colorectal Adenomatous Polyposis. Am. J. Hum. Genet..

[7] Tseng-Rogenski S.S., Chung H., Wilk M.B., Zhang S., Iwaizumi M., Carethers J.M. (2012). Oxidative stress induces nuclear-to-cytosol shift of hMSH3, a potential mechanism for EMAST in colorectal cancer cells. PLoS ONE.

[8] Tseng-Rogenski S.S., Hamaya Y., Choi D.Y., Carethers J.M. (2015). Interleukin 6 alters localization of hMSH3, leading to DNA mismatch repair defects in colorectal cancer cells. Gastroenterology.

[9] Nakajima E., Orimo H., Ikejima M., Shimada T. (1995). Nine-bp repeat polymorphism in exon 1 of the hMSH3 gene. Jpn. J. Hum. Genet..

[10] Tseng-Rogenski S.S., Munakata K., Choi D.Y., Martin P.K., Mehta S., Koi M., Zheng W., Zhang Y., Carethers J.M. (2020). The Human DNA Mismatch Repair Protein MSH3 Contains Nuclear Localization and Export Signals That Enable Nuclear-Cytosolic Shuttling in Response to Inflammation. Mol. Cell. Biol..

[11] Miao H.K., Chen L.P., Cai D.P., Kong W.J., Xiao L., Lin J. (2015). MSH3 rs26279 polymorphism increases cancer risk: A meta-analysis. Int. J. Clin. Exp. Pathol..

[12] Holmer R., Wätzig G.H., Tiwari S., Rose-John S., Kalthoff H. (2015). Interleukin-6 trans-signaling increases the expression of carcinoembryonic antigen-related cell adhesion molecules 5 and 6 in colorectal cancer cells. BMC Cancer.

[13] Rose-John S., Jenkins B.J., Garbers C., Moll J.M., Scheller J. (2023). Targeting IL-6 trans-signalling: Past, present and future prospects. Nat. Rev. Immunol..

[14] Taher M.Y., Davies D.M., Maher J. (2018). The role of the interleukin (IL)-6/IL-6 receptor axis in cancer. Biochem. Soc. Trans..

[15] Jostock T., Müllberg J., Ozbek S., Atreya R., Blinn G., Voltz N., Fischer M., Neurath M.F., Rose-John S. (2001). Soluble gp130 is the natural inhibitor of soluble interleukin-6 receptor transsignaling responses. Eur. J. Biochem..

[16] Terry C.F., Loukaci V., Green F.R. (2000). Cooperative influence of genetic polymorphisms on interleukin 6 transcriptional regulation. J. Biol. Chem..

[17] Jones K.G., Brull D.J., Brown L.C., Sian M., Greenhalgh R.M., Humphries S.E., Powell J.T. (2001). Interleukin-6 (IL-6) and the prognosis of abdominal aortic aneurysms. Circulation.

[18] Garbers C., Monhasery N., Aparicio-Siegmund S., Lokau J., Baran P., Nowell M.A., Jones S.A., Rose-John S., Scheller J. (2014). The interleukin-6 receptor Asp358Ala single nucleotide polymorphism rs2228145 confers increased proteolytic conversion rates by ADAM proteases. Biochim. Biophys. Acta.

[19] Wonnerth A., Katsaros K.M., Krychtiuk K.A., Speidl W.S., Kaun C., Thaler K., Huber K., Wojta J., Maurer G., Seljeflot I. (2014). Glycoprotein 130 polymorphism predicts soluble glycoprotein 130 levels. Metabolism.

[20] Spaventi R., Pecur L., Pavelic K., Pavelic Z.P., Spaventi S., Stambrook P.J. (1994). Human tumour bank in Croatia: A possible model for a small bank as part of the future European tumour bank network. Eur. J. Cancer.

[21] Boland C.R., Thibodeau S.N., Hamilton S.R., Sidransky D., Eshleman J.R., Burt R.W., Meltzer S.J., Rodriguez-Bigas M.A., Fodde R., Ranzani G.N. (1998). A National Cancer Institute Workshop on Microsatellite Instability for cancer detection and familial predisposition: Development of international criteria for the determination of microsatellite instability in colorectal cancer. Cancer Res..

[22] Hanahan D., Weinberg R.A. (2021). Hallmarks of cancer: The next generation. Cell.

[23] Hanahan D. (2022). Hallmarks of Cancer: New Dimensions. Cancer Discov..

[24] Becker C., Fantini M.C., Wirtz S., Nikolaev A., Lehr H.A., Galle P.R., Rose-John S., Neurath M.F. (2005). IL-6 signaling promotes tumor growth in colorectal cancer. Cell Cycle.

[25] Waldner M.J., Foersch S., Neurath M.F. (2012). Interleukin-6-a key regulator of colorectal cancer development. Int. J. Biol. Sci..

[26] Munakata K., Koi M., Kitajima T., Tseng-Rogenski S., Uemura M., Matsuno H., Kawai K., Sekido Y., Mizushima T., Toiyama Y. (2019). Inflammation-Associated Microsatellite Alterations Caused by MSH3 Dysfunction Are Prevalent in Ulcerative Colitis and Increase With Neoplastic Advancement. Clin. Transl. Gastroenterol..

[27] Mas-Ponte D., McCullough M., Supek F. (2022). Spectrum of DNA mismatch repair failures viewed through the lens of cancer genomics and implications for therapy. Clin. Sci..

[28] Koi M., Tseng-Rogenski S.S., Carethers J.M. (2018). Inflammation-associated microsatellite alterations: Mechanisms and significance in the prognosis of patients with colorectal cancer. World J. Gastrointest. Oncol..

[29] IL6R Genetics Consortium Emerging Risk Factors Collaboration (2012). Interleukin-6 receptor pathways in coronary heart disease: A collaborative meta-analysis of 82 studies. Lancet.

[30] Atreya R., Mudter J., Finotto S., Müllberg J., Jostock T., Wirtz S., Schütz M., Bartsch B., Holtmann M., Becker C. (2000). Blockade of interleukin 6 trans signaling suppresses T-cell resistance against apoptosis in chronic intestinal inflammation: Evidence in Crohn disease and experimental colitis in vivo. Nat. Med..

